# Acquired hemophilia A as a disease of the elderly: A comprehensive review of epidemiology, pathogenesis, and novel therapy

**DOI:** 10.1007/s11357-024-01317-7

**Published:** 2024-09-23

**Authors:** Andrea Lehoczki, Mónika Fekete, Gábor Mikala, Imre Bodó

**Affiliations:** 1 Doctoral College, Health Sciences Program, Semmelweis University, Budapest, Hungary; 2 Institute of Preventive Medicine and Public Health, Semmelweis University, Budapest, Hungary; 3 Departments of Hematology and Stem Cell Transplantation, South Pest Central Hospital, National Institute of Hematology and Infectious Diseases, Szent László Campus, Budapest, Hungary; 4 Department of Internal Medicine and Hematology, Semmelweis University, Budapest, Hungary; 5 Department of Hematology and Medical Oncology, Emory University, Atlanta, GA USA

**Keywords:** Acquired hemophilia A, AHA, Coagulopathy, Autoimmune, Autoimmunity, Aging, Age-related, Autoantibody, Bleeding

## Abstract

Acquired hemophilia A (AHA) is a rare autoimmune bleeding disorder characterized by the development of neutralizing autoantibodies (inhibitors) against coagulation factor VIII (FVIII). This review provides an in-depth exploration of AHA, covering its epidemiology, pathogenesis, clinical presentation, diagnosis, complications, and treatment strategies, focusing on recent advancements. AHA can manifest in both men and women with no prior bleeding history. The annual incidence is estimated to be approximately 1 case per million individuals in the general population. The incidence increases significantly with age: the incidence among individuals aged 60 years or older is approximately 3 to 4 cases per million individuals per year. Typically, patients present with an acquired bleeding disorder that is characterized by an isolated, prolonged activated partial thromboplastin time stemming from FVIII deficiency. Diagnosis relies on the detection of neutralizing antibodies using the Nijmegen-modified Bethesda assay. Hemostatic control strategies involve bypassing agents like recombinant activated factor VII, activated prothrombin complex concentrate, and recombinant porcine FVIII for bleeding patients. Emicizumab, a novel bypassing agent, exhibits several potential advantages. In the realm of immunosuppressive treatment for inhibitor eradication, the CyDRi regimen emerged as a remarkable advancement, significantly enhancing the outlook for the management of AHA even in the elderly frail population.

## Introduction

Acquired hemophilia A (AHA) has been identified since the 1940s [1]. It is a rare and potentially life-threatening bleeding disorder caused by autoantibodies against coagulation factor VIII (FVIII), which causes increased clearance and neutralization of FVIII [2]. Unlike congenital hemophilia A which is inherited and typically manifests in early childhood, AHA occurs in individuals with no previous personal or family history of bleeding. AHA is associated with significant morbidity and mortality unless promptly diagnosed and managed appropriately [2].

The challenge of managing AHA lies in its unique clinical and immunological characteristics. These inhibitory antibodies interfere with the effectiveness of FVIII replacement therapy, making management of bleeding episodes difficult. Additionally, AHA is often underdiagnosed or misdiagnosed due to its rarity and the tendency at some institutions to only order PT/INR to evaluate the hemostasis, resulting in delayed initiation of appropriate treatment and potentially life-threatening consequences [3, 4].

In this comprehensive review, we aim to explore the epidemiology, pathogenesis, clinical manifestations, and recent breakthroughs in the management strategies of AHA. A better understanding of AHA will facilitate early recognition, inform clinical decision-making, improve patient outcomes, and pave the way for further research and advancements in the field.

### Comparison with congenital hemophilia A and congenital hemophilia A with inhibitors

For the purpose of this review, it is important to briefly compare AHA with congenital hemophilia A for a thorough understanding of these two forms of hemophilia. While congenital hemophilia A results from mutations in the FVIII gene and is a lifelong condition, AHA—an autoimmune coagulation disorder—occurs due to the development of inhibitory autoantibodies against FVIII in individuals without a genetic predisposition. Individuals with congenital hemophilia A also have the potential to develop inhibitors, which are IgG alloantibodies directed against exogenous therapeutic FVIII [5–7]. These antibodies effectively counteract the function of infused clotting factor concentrates [5–7]. Understanding the differences between congenital hemophilia A and AHA is essential for accurate diagnosis, tailored management, and prognostic considerations. Significant differences between congenital hemophilia A and AHA lie in the age of onset, the clinical bleeding manifestations of the two diseases, and the types of therapeutic management strategies.

Congenital hemophilia A, unlike AHA, is characterized by a deficiency of FVIII from birth in male infants. Congenital hemophilia A is generally diagnosed in infancy or early childhood, whereas AHA can occur at any age, but typically presents in the peripartum period, or at later ages in both genders. In terms of prevalence, congenital hemophilia A is more common than AHA. Congenital hemophilia A has an estimated prevalence of approximately 1 in 5000 male births, while the prevalence of AHA is only 1.5 per million inhabitants.

Congenital hemophilia A is classified into three severity levels based on FVIII activity levels: severe (< 1% FVIII activity), moderate (1–5% FVIII activity), and mild (6–40% FVIII activity). The severity of congenital hemophilia A determines the frequency and severity of spontaneous bleeding episodes, such as joint bleeds, muscle bleeds, or prolonged bleeding after injuries or surgeries. In contrast to congenital hemophilia that reliably follows the above factor level-phenotype rule, the severity and manifestation of bleeding in AHA can vary widely and may result in sudden and unpredictable bleeding episodes independent of actual FVIII levels. The types of bleeding associated with AHA include soft tissue hematomas, muscle bleeds, mucosal bleeding, gastrointestinal bleeding, genitourinary bleeding, intracranial hemorrhage, and prolonged bleeding after injuries or surgeries, but joint bleeds—the most common bleeding type in hereditary hemophilia—are infrequent [8–19]. The physiological explanations behind these phenotypic differences are incompletely understood.

The management and treatment strategies to eradicate inhibitors for congenital hemophilia A with inhibitory alloantibodies and AHA also differ. While replacement immune tolerance induction (ITI) using high-dose therapy with FVIII concentrates is the mainstay of treatment for inhibitors in congenital hemophilia A, treatment for AHA has traditionally included immunosuppressive agents. At the same time, bypassing agents have been used in both conditions to stop bleeding and to achieve hemostatic control.

## Epidemiology of AHA

### Prevalence and incidence rates

AHA is considered a rare bleeding disorder, with an estimated prevalence ranging from 1 to 4 cases per million individuals. However, due to its underdiagnosis and misdiagnosis, the actual prevalence may be higher. The incidence rates of AHA vary across different populations and age groups. In the general population, the annual incidence is estimated to be around 1 to 1.78 cases per million individuals [20–22]. However, the incidence increases significantly with age, particularly in the elderly population [2, 21, 23]. The incidence among individuals aged 60 years or older has been reported to be approximately 3 to 4 cases per million individuals annually. This age-related increase in incidence may be attributed to age-related changes in the immune system and the presence of comorbidities.

### Age and sex distribution

AHA can occur at any age, from neonates to the elderly [22, 24, 25]. In general, the age distribution of AHA follows a bimodal pattern. The first peak occurs in individuals younger than 30 years, which is predominantly associated with pregnancy-related cases. The second peak occurs in older individuals with a median age at diagnosis around 75 [2, 21, 23, 26], which is often associated with autoimmune and malignant disorders [2]. Regarding sex distribution, AHA affects both males and females [2]. However, some studies have reported a slight male predominance, with a male-to-female ratio of around 1.2:1 [2]. The reasons behind this male preponderance are not yet fully understood but could be attributed to hormonal and immunological factors.

### Associated risk factors and predisposing conditions

AHA can occur spontaneously or in association with various underlying conditions. Approximately 50 to 80% of AHA cases are idiopathic, meaning no underlying disease can be identified [2, 21]. However, several risk factors and predisposing conditions have been recognized, including autoimmune disorders, malignancies, pregnancy, and certain medications.

#### Autoimmune disorders

AHA has been associated with autoimmune diseases [27–29], such as rheumatoid arthritis [30], systemic lupus erythematosus [31], and autoimmune thyroid diseases. The immune dysregulation observed in these conditions may contribute to the development of inhibitory antibodies against FVIII and is responsible for approximately 10% of AHA cases [2]. A recent population-based study has shed light on an intriguing finding: comorbid Alzheimer’s disease appears to be a significant risk factor for AHA [30, 32]. This discovery adds a new layer of evidence to the growing body of research supporting the concept that Alzheimer’s disease may have autoimmune components.

The age predilection observed in AHA mirrors trends seen in other autoimmune disorders like rheumatoid arthritis [33, 34]. This phenomenon is thought to stem from multifaceted mechanisms related to aging and immune function [35, 36]. Age-related changes such as alterations in T-cell function, compromised regulatory mechanisms, and heightened susceptibility to autoimmune responses due to cumulative antigen exposure over a lifetime are believed to play pivotal roles [35, 37–41]. Additionally, dysbiosis, characterized by changes in the composition and function of the microbiota with age, has also been implicated in promoting autoimmune diseases among older adults [42, 43]. These insights underscore the intricate interplay between aging and immune dysregulation in the pathogenesis of autoimmune disorders, including AHA.

#### Malignancies

Hematological malignancies, including lymphoproliferative disorders [44] and solid tumors (such as prostate, lung, and gastrointestinal cancers), have been linked to AHA, accounting for approximately 10% of all cases [2, 45]. The tumor microenvironment and aberrant immune responses may play a role in the development of inhibitory antibodies [46–51].

#### Pregnancy and postpartum period

AHA can occur during pregnancy or the postpartum period [52–56]. The exact mechanisms underlying this association remain unclear, but changes in the maternal immune system during pregnancy may contribute to the development of inhibitory antibodies that contribute to 2 to 15% of AHA cases [57].

#### Medications and drug reactions

Certain medications, such as penicillins, cephalosporins, and interferons, have been implicated in the development of AHA in a subset of cases, ranging from 3 to 5% [58–63]. These drug-induced immune reactions may trigger the production of inhibitory antibodies against FVIII. A recent global pharmaco-epidemiologic study has compiled a list of 14 drugs that have been linked to AHA [64]. This list includes commonly used medications such as the antiplatelet agent clopidogrel as well as alemtuzumab (a monoclonal antibody used primarily in the treatment of certain types of leukemia, particularly chronic lymphocytic leukemia, and multiple sclerosis) and omalizumab (a monoclonal antibody used to treat allergic asthma) [64]. Notably, the median age at which drug-induced AHA was reported to onset is 75 years, and the median time from the initiation of the suspected drug to the development of AHA symptoms was only 30 days [64]. Tragically, 10% of cases resulted in fatality [64]. Additionally, a recent systematic review has identified 19 cases of AHA that developed following anti-SARS-CoV-2 vaccination. These cases predominantly occurred in elderly male patients with multiple underlying health conditions after receiving mRNA vaccines, specifically BNT162b2 Pfizer-BioNTech and mRNA-1273 Moderna [65]. As with other purported consequences of vaccination, the cause-effect relationship has yet to be proven.

## Pathogenesis of AHA

The pathogenesis of AHA is of a typical autoimmune nature, in which the immune system mistakenly recognizes FVIII as a foreign antigen and mounts an immune response against it. This autoimmune response leads to the production of inhibitory antibodies, also known as inhibitors, that specifically target FVIII and neutralize its activity and increase its clearance.

The exact mechanisms involved in the development of AHA are not fully elucidated, but several hypotheses have been proposed. One hypothesis suggests that AHA arises from the breakdown of immune tolerance to FVIII. Under normal circumstances, the immune system tolerates self-antigens, including FVIII, to prevent autoimmunity. However, in AHA, there is a failure in immune tolerance mechanisms, leading to the production of inhibitory antibodies.

Inhibitory antibodies are central to the pathogenesis of AHA. These antibodies bind to FVIII and inhibit its normal clotting function. FVIII plays a crucial role as a cofactor within the tenase complex in which factor IX (FIX) converts factor X (FX) to Xa [66], leading to the generation of thrombin on the surface of activated platelets. FVIII itself is initially a large precursor protein (330 kDa). Through proteolytic processing, it undergoes structural changes, converting into heterodimers of heavy and light chains, altering its domain structure from A1-a1-A2-a2-B-a3-A3-C1-C2 [66]. In AHA, the majority of inhibitors targeting FVIII bind to specific domains, namely A2, A3, or C2 [59, 67–69]. The A2 and A3 domains are responsible for FVIII’s interaction with factors IXa and X, whereas the C2 domain binds it to phospholipids and von Willebrand factor.

Autoantibodies produced in AHA patients are typically polyclonal and belong to the IgG class, primarily IgG1 and IgG4 [59, 67–69]. Similarly, alloantibodies observed in congenital hemophilia A patients with inhibitors also predominantly fall under the IgG1 and IgG4 subclasses. Intriguingly, both auto- and alloantibodies tend to target the same regions on the FVIII molecule (A2, A3, and C2 domains), disrupting its interaction with activated factor IX, phospholipids, and von Willebrand factor [59, 67–69]. This results in insufficient production of thrombin on the surface of activated platelets [66].

The most notable contrast between auto- and alloantibodies lies in their inactivation patterns. In congenital hemophilia, in patients who receive exogenous FVIII, most inhibitors are categorized as “type 1,” resulting in linear inactivation when plotted against the inhibitor’s plasma concentration [59, 67–69]. These antibodies can completely neutralize FVIII when present in high concentrations [59, 67–69]. Conversely, most autoantibodies in AHA are “type 2,” displaying a non-linear, complex inactivation pattern [59, 67–69]. These antibodies bind to FVIII domains in a time and temperature-dependent manner, following second-order kinetics, significantly reducing FVIII activity [59, 67–69]. While some residual FVIII activity may remain, it is not clinically protective, potentially leading to an underestimation of the inhibitor’s potency [59, 67–69].

The presence of inhibitory antibodies poses a significant challenge in the clinical management of AHA. Inhibitors reduce the effectiveness of FVIII replacement therapy, making it difficult to achieve hemostatic control during bleeding episodes. Treatment strategies aim to bypass the inhibitory effect of antibodies and restore hemostasis through alternative clotting factor replacement approaches.

## Clinical presentation and diagnosis

### Symptoms and bleeding patterns

The clinical presentation of AHA can vary widely, depending on the severity of the FVIII deficiency and the location of bleeding [2, 14, 22, 25, 70, 71]. One of the primary challenges associated with AHA is a pervasive lack of awareness, which often leads to a delay in diagnosis and the initiation of treatment. In many cases, patients initially present in emergency settings to healthcare professionals who may not specialize in managing bleeding disorders. This diagnostic delay is a significant concern, with data from the European Acquired Hemophilia Registry (EACH2) revealing that 34% of patients experienced a delay of 1 week or more between the onset of bleeding symptoms and the definitive diagnosis and initiation of treatment [2]. It is paramount to recognize the symptoms and bleeding patterns associated with this condition for timely diagnosis and intervention.

Common symptoms of AHA include spontaneous or prolonged bleeding, often in soft tissues such as the skin, muscles, or mucous membranes. Patients may experience easy bruising, hematoma formation, prolonged bleeding from minor cuts or injuries, and excessive bleeding after surgical procedures. In severe cases, spontaneous and life-threatening bleeding episodes, such as gastrointestinal or intracranial hemorrhages, may occur. Roughly 10% of patients do not exhibit bleeding symptoms; hence, a prolonged activated partial thromboplastin time (aPTT) should always be investigated before invasive procedures [72].

### Laboratory findings and hemostatic assessments

Diagnosing AHA requires a combination of clinical evaluation, laboratory tests, and specific hemostatic assessments [72]. Isolated prolongation of aPTT is the laboratory hallmark of AHA due to the inhibitory effect of the circulating antibodies on FVIII. Mixing studies involve 1:1 mixing patient plasma with normal plasma and assessing its consequences on the aPTT immediately and after a 2-h incubation at 37 °C. In AHA, the prolonged aPTT activity does not correct after mixing, confirming the presence of an inhibitor. Given the type 2 kinetics of most AHA inhibitors, non-correction by the addition of normal plasma is most obvious in the 2-h sample.

Measurement of FVIII activity is informative for confirming the diagnosis. AHA typically presents with significantly reduced FVIII activity levels (< 0.3 IU/dL, often < 0.01 IU/dL), and FIX, FXI, and FXII as well as vWF levels are not significantly reduced, reflecting the monospecific nature of the inhibitory antibodies.

The Bethesda assay, initially designed to identify and quantify FVIII alloantibodies in congenital hemophilia A, also finds utility in detecting FVIII inhibitors in AHA [72]. The Nijmegen-modified Bethesda assay measures residual FVIII activity after mixing a series of dilutions of normal plasma with patient plasma. The results are reported as Bethesda units (BU/mL) that reflect the dilution with 0.5 IU/dL residual FVIII activity, i.e., higher values indicating higher inhibitor concentrations. However, due to the often complex and non-linear type 2 kinetics displayed by FVIII inhibitors in AHA, the Bethesda assay may not be an ideal estimation of autoantibody’s inhibitory potential [72].

### Differential diagnosis

AHA should be distinguished from congenital hemophilia A which is an inherited disorder. History is usually the best tool in demonstrating the acquired nature of the bleeding diathesis. A careful evaluation of the patient’s personal and family bleeding history, as well as plasma mixing studies, can help differentiate between the two. Genetic testing is rarely needed but may occasionally aid in managing family members in case of hereditary hemophilia.

von Willebrand disease (vWD) can sometimes present with similar clinical features as AHA. Laboratory testing for vWD, including von Willebrand factor antigen and activity levels, can aid in distinguishing between the two conditions. Spontaneous autoantibody inhibitors to other coagulation factors within the intrinsic pathway are exceedingly rare, and measuring FIX, FXI, and FXII factor levels will render the diagnosis clear. Acquired von Willebrand syndrome (AVWS) is a realistic differential diagnostic consideration. Measuring von Willebrand factor antigen and activity levels is essential in making the correct diagnosis as some AVWS patients may have very low vWF levels with consequentially reduced FVIII and aPTT prolongation.

Other acquired coagulopathies, such as liver disease, disseminated intravascular coagulation (DIC), or vitamin K deficiency, can also present with bleeding symptoms. An assessment of liver function, coagulation profile, and additional laboratory tests can assist in ruling out these conditions.

Platelet disorders, such as immune thrombocytopenic purpura (ITP) or platelet function defects, can rarely mimic AHA. Evaluation of platelet counts, platelet function tests, and clinical assessment can help differentiate between these conditions.

## Complications and consequences of *AHA*

### Association with morbidity and mortality

AHA is associated with significant morbidity and mortality if not promptly diagnosed and managed appropriately [25]. The mortality rate for AHA can be as high as 22% [20, 67, 73, 74]. Uncontrolled bleeding episodes can lead to severe complications, such as compartment syndromes from deep muscle hematomas, and life-threatening internal bleeding, including gastrointestinal or intracranial hemorrhages. In addition, FVIII inhibitors complicate treatment and increase the risk of treatment failure during bleeding episodes explaining the need for intensive hemostatic control with special bypass agents. The potential for delayed recognition of AHA further contributes to increased morbidity and mortality rates in affected individuals.

### Impact on quality of life

The general timeframe for eradication of inhibitors in AHA can vary widely among individuals and depends on various factors, including the severity of the condition, the effectiveness of the chosen treatment regimen, and the presence of any underlying medical conditions. In some cases, inhibitors may be eradicated within a few weeks or months of initiating treatment, while in others, it may take several months or even years to achieve successful eradication. Additionally, some patients may never fully eradicate inhibitors despite ongoing treatment efforts. AHA can profoundly impact the quality of life for those affected, particularly if efforts to eradicate autoantibodies are unsuccessful for an extended time. While the rarity of the disease precludes systematic studies, our conclusions draw from extensive clinical experience at a major national hematology center. Patients with persisting inhibitors commonly experience anxiety about bleeding and its potential complications.

## Management and treatment strategies

The management of AHA requires a multidisciplinary approach. The primary goals of treatment are to control bleeding episodes and to eradicate inhibitory antibodies thereby restoring hemostasis.

### Hemostatic control and bleeding management

Achieving hemostatic control is crucial in the management of AHA [75]. FVIII replacement therapy is expected to be ineffective due to the presence of inhibitors and can only be attempted for low inhibitor titers. Even high doses of FVIII may not always have the power to overcome the inhibitory effect.

Porcine FVIII is active in human hemostasis but is distinctly different for several epitopes targeted by anti-FVIII autoantibodies. Therefore, another therapeutic option is porcine FVIII, therapy, for which determining anti-porcine inhibitor titers can be highly valuable. Decades ago, porcine plasma-derived FVIII treatment was successfully employed in clinical practice. It has been removed from the market, but since the 2000s, it has been available as a recombinant product, rpFVIII. Local hemostatic measures, such as compression, suturing, and topical hemostatic agents, can be utilized to control bleeding from superficial wounds or mucosal surfaces.

Classical bypassing agents, such as activated prothrombin complex concentrate (aPCC) and recombinant activated factor VII (rFVIIa), are used to bypass the need for FVIII and restore hemostasis [76, 77]. These agents provide FVIII-independent pathways for clot formation either by a pharmacological concentration of FVIIa, which then gives rise to a large primary thrombin surge, or by using activated common pathway (primarily Xa) factors to forgo the need for the tenase complex.

#### Emicizumab for bleeding prophylaxis

Previously, prophylactic FEIBA was the primary approach employed in patients with AHA until inhibitor eradication was achieved [78]. Lately, a new option has emerged in the form of emicizumab, a bispecific monoclonal antibody that mimics the function of FVIII [79, 80]. Emicizumab which has demonstrated efficacy in treating congenital hemophilia A with or without inhibitors, is now under investigation for its potential in managing AHA [81–85]. Early studies have shown promising results, particularly in achieving hemostatic control and reducing the frequency of bleeding episodes [81–85]. These reports primarily discuss the off-label use of emicizumab for short- or long-term prophylaxis, even before inhibitor eradication has occurred. However, in some cases, it has been successfully employed as an additional measure for acute bleeding control, demonstrating its versatility in AHA management, notably in studies like AGEHA.

At present, specific recommendations regarding the use of emicizumab in AHA, both in terms of indications and potential side effects, are yet to be established. Nevertheless, one notable advantage of emicizumab is its subcutaneous administration, which sets it apart from other products requiring intravenous administration. Additionally, its dosing frequency offers further convenience, making it a potential candidate for at-home administration. However, it is essential to highlight the potential risk of thromboembolic complications associated with emicizumab [85]. Therefore, close monitoring of FVIII activity is crucial. If, as a result of the immunosuppressive treatment, FVIII levels reach or surpass 50%, emicizumab treatment must be promptly discontinued.

### Immunosuppressive therapies and inhibitor eradication

Immunosuppressive therapies represent the cornerstone of AHA treatment, aiming to eliminate inhibitory antibodies. Achieving remission, defined as the absence of bleeding episodes and sustained normalization of FVIII levels without the need for bypassing agents, is a primary goal of treatment. Remission induction can be challenging, especially in cases with high-titer or persistent inhibitors. Until recently, managing AHA was a complex challenge, and the optimal approach remained elusive. First-line immunosuppressive regimens included steroid monotherapy, steroid combinations with cyclophosphamide, or steroids combined with rituximab—a monoclonal antibody designed to target CD20, a B-cell antigen responsible for autoantibody production. These treatment approaches achieved a success rate of approximately 50 to 83% [24, 70, 86]. Notably, cases with higher anti-FVIII antibody titers tended to have lower rates of complete remission [87, 88]. In instances of treatment failure, secondary agents like cyclosporine, rituximab, and others were employed. However, it is essential to acknowledge that these immunosuppressive therapy protocols were associated with substantial toxicity and posed mortality risks, particularly for frail elderly patients.

Recurrence of AHA can occur even after successful treatment and remission. Vigilance is necessary, particularly in patients with underlying conditions, as the inhibitor may reappear or new inhibitors may develop.

#### Combined immunosuppression for *AHA* using the highly effective low-toxicity CyDRi regimen

We recently reported a significant breakthrough in the treatment of AHA. Our study introduced the upfront combined CyDRi regimen, which employs pulse doses of cyclophosphamide, dexamethasone, and low-dose rituximab [89]. Notably, this regimen has demonstrated rapid efficacy, especially in elderly AHA patients, with remarkably low toxicity and impressive overall survival rates [89]. The CyDRi regimen comprises cyclophosphamide administered at 1000 mg on days 1 and 22; dexamethasone at 40 mg on days 1, 8, 15, and 22; and rituximab at 100 mg on days 1, 8, 15, and 22 [89]. What sets this treatment approach apart from the previous ones are several novel aspects: the use of dexamethasone as the steroid component, the upfront combination of all three drugs, the pulse dosing schedule for each of them, and the consistent use of this regimen even for cases of resistant or relapsed disease. Remarkably, the CyDRi regimen has yielded significantly higher complete remission rates, reaching an impressive 96.8% and a remarkable overall survival rate of 90.6%, surpassing those achieved with currently used sequential regimens [89]. The regimen’s favorable toxicity profile has played a pivotal role in these remarkable outcomes. The CyDRi regimen emerges as an attractive and effective option for immune suppression therapy in elderly patients with AHA and holds the potential to influence future treatment guidelines.

## Conclusions

Due to the rarity of AHA, large-scale randomized trials remain a challenge, leaving room for continued exploration in determining the optimal algorithm for both bleeding prophylaxis and immunosuppressive therapy. Nevertheless, emicizumab has emerged as a promising addition to the prophylactic toolkit [79–81, 83–85]. In 2022, the CyDRi protocol presented a well-tolerated and highly effective immunosuppressive approach, offering a notable advancement in AHA management even in the elderly frail population [89]. The development of improved consensus guidelines and standardized treatment protocols is crucial to harmonize patient care and establish consistent practices across various healthcare settings [72]. Early diagnosis and intervention are pivotal in improving AHA outcomes. Further research should explore targeted screening strategies, particularly in high-risk populations or individuals with autoimmune conditions or malignancies. This approach may help identify cases of AHA at an earlier stage, allowing for prompt intervention and potentially better prognoses. Lastly, raising awareness among healthcare professionals regarding the clinical presentation and diagnostic complexities associated with AHA is imperative. By improving recognition and facilitating specialized care referrals, we can ensure that AHA patients receive the timely and tailored treatments they require.
